# Mapping Current Fields in a Bay Using a Coast-Fitting Tomographic Inversion

**DOI:** 10.3390/s20020558

**Published:** 2020-01-20

**Authors:** Minmo Chen, Ze-Nan Zhu, Chuanzheng Zhang, Xiao-Hua Zhu, Min Wang, Xiaopeng Fan, Ruixiang Zhao, Ju Lin, Arata Kaneko

**Affiliations:** 1 State Key Laboratory of Satellite Ocean Environment Dynamics, Second Institute of Oceanography, Ministry of Natural Resources, Hangzhou 310012, China; mmchen_t9@163.com (M.C.); zzn@sio.org.cn (Z.-N.Z.); zhangcz@sio.org.cn (C.Z.); wangm0309@163.com (M.W.); xiaopengfan@163.com (X.F.); zhaorx@sio.org.cn (R.Z.); 2 Southern Marine Science and Engineering Guangdong Laboratory (Zhuhai), Zhuhai 519000, China; 3 College of Information Science & Engineering, Ocean University of China, Qingdao 266100, China; julin97@gmail.com; 4 Graduate School of Engineering, Hiroshima University, Higashi-Hiroshima, Hiroshima 739-8527, Japan; akaneko@hiroshima-u.ac.jp

**Keywords:** coastal acoustic tomography, coast-fitting tomographic inversion, semidiurnal tidal current, residual current, current-field mapping, net volume transport

## Abstract

Coast-fitting tomographic inversion that is based on function expansion using three types of normal modes (the Dirichlet, Neumann, and open boundary modes) is proposed to reconstruct current fields from the coastal acoustic tomography (CAT) data. The superiority of the method was validated while using CAT data that were obtained in 2015 in the Dalian Bay. The semidiurnal tidal and residual current fields were accurately reconstructed over the entire model domain surrounded by coasts and open boundaries. The proposed method was effective, particularly around the peripheral regions of the tomography domain and the near-coast regions outside the domain, where accurate results are not expected from the conventional inverse method based on function expansion by Fourier function series with no coast fittings. The error velocity for the semidiurnal tidal currents was 2.2 cm s^−1^, which was calculated from the root-mean-square-difference between the CAT-observed and inverted range-averaged currents that were obtained along the nine peripheral transmission paths. The error velocity for the residual currents estimated from the 12-h mean net residual transport at the bay mouth was 0.9 cm s^−1^. The errors were significantly smaller than the amplitude of the tidal and residual currents.

## 1. Introduction

Coastal acoustic tomography (CAT), which was developed as a coastal-sea application for ocean acoustic tomography [1,2], still serves as innovative technology for monitoring and predicting variations in the coastal sea environment by data assimilation [3,4,5,6]. Mapping rapidly-varying current fields in coastal seas is a remarkable capability of CAT [7,8,9,10,11,12,13]. A CAT experiment with a horizontal resolution of 1.53 km was conducted in 2015 in the Dalian Bay, China, in which 51 sound transmission paths were constructed for 11 CAT stations [14]. The current fields were reconstructed while using conventional tomographic inversion based on Fourier function expansion with no coast constraints. The inverted currents provided a precise result, indicating that the root-mean-square-differences (RMSDs) with the one-point moored acoustic Doppler current profiler data were 4.04 cm s^−1^ and 3.80 cm s^−1^ for the eastward and northward currents, respectively.

In this study, tomographic inversion that is based on function expansion using three types of coast-fitting normal modes, namely the Dirichlet, Neumann, and open boundary modes, is proposed for improving the current fields reconstructed while using conventional tomographic inversion of the 2015 Dalian Bay data. Special attention is paid to the peripheral regions of the tomography domain and the near-coast regions outside the domain.

## 2. Methods

This study used the reciprocal sound transmission data that were obtained in the 2015 Dalian Bay tomography experiment to confirm the superiority of the proposed coast-fitting inverse method as compared with the results from the conventional inverse method with no coast constraints. The proposed and conventional methods are referred to as the coast-fitting method (CFM) and the no coast method (NCM), respectively. A detailed explanation of the site and the experimental conditions can be found in the study by Zhang et al. [14]. The NCM is based on the Fourier function expansion of two-dimensional current fields in the tomography domain and the tapered least squares method determined the expansion coefficients. Any effects from the coastlines surrounding the tomography domain are not taken into account in the inversion. The procedure for the conventional inversion method can also be found in the above study. The 2-min. interval sound transmission and the hourly moving average of the original received data used for data processing are useful parameters in this study.

**Figure 1:**
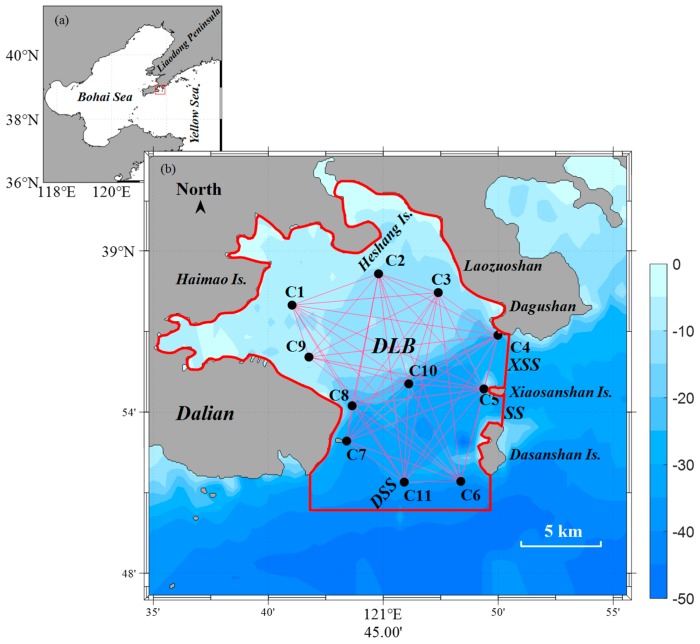
(**a**) Location map of the Dalian Bay. (**b**) Model domain of the 2015 Dalian Bay data analysis encircled with thick red line. The 11 acoustic stations (C1–C11) are indicated with black dots and the transmission paths to each station are indicated with thin cyan lines. The southern inlet and southeastern inlets are labelled as DSS, and XSS, and SS, respectively. The 5-km scale is indicated at the lower right corner.

The model domain is the region that is surrounded by the coastlines on the northern and western sides and the open boundaries at the southern and southeastern inlets of the bay, being labelled as DSS, XSS, and SS in Figure 1. The tomography domain is defined as the domain encircled by the 10 peripheral transmission paths of C1C2, C2C3, C3C4, C4C5, C5C6, C6C11, C11C7, C7C8, C8C9, and C9C11 connecting the neighboring acoustic stations; however, data were not obtained between C8 and C9 due to the protruded breakwater. The open boundaries were located offshore by 1–5 km from the peripheral transmission paths at the bay mouth. The number of the successful reciprocal transmission data reached 51 for 14 h from 20:00 on 7 March to 10:00 on 8 March 2015. The semidiurnal tidal and residual currents were studied while using the 12-h data from 20:00 on 7 March to 08:00 on 8 March 2015.

The current, *v_j_* at the *j*th grid along an acoustic ray projected to a horizontal slice is related to the differential travel time, Δτ of the acoustic ray, as follows [1]:

(1) $$\Delta \tau =-2\int_{0}^{L}\frac{v_{j}}{C_{0}^{2}}d\xi ,$$

where *L* denotes the total length of the ray and dξ denotes the segmented length of the ray. As typically found in the paper by Zhang et al. [13], the current fields in Equation (1) are conventionally expanded into the Fourier function series, in which the first expansion term has the wavelength twice the size of the tomography domain. However, no constraints can be considered at the coast in the NCM. A CFM is proposed in this study while using the three normal mode fitting functions of the coastlines. The proposed method is derived from the normal mode function expansion method for interpolating and filtering the current field data that were obtained in the near-coast regions while using high-frequency ocean radars [15,16,17]. The two-dimensional current fields are decomposed into of the Dirichlet, Neumann, and open-boundary modes,

(2) $$v=\sum_{i=1}^{\infty }\alpha_{i}^{\psi }k\times \nabla \psi_{i}+\sum_{i=1}^{\infty }\alpha_{i}^{\phi }\nabla \phi_{i}+\sum_{i=1}^{\infty }\alpha_{i}^{b}\nabla \phi_{i}^{b},$$

where $\psi_{i}$ and $\phi_{i}$ denote the stream and potential functions of the *i*th mode, respectively. $\phi_{i}^{b}$ denotes the open-boundary mode of the *i*th mode and ***k*** denotes the unit vector orthogonal to the horizontal plane. The first and second terms of Equation (2) represent the solenoidal and irrotational components in a two-dimensional current field, respectively.

The term, $\psi_{i}$ is numerically determined by solving the two-order partial differential equation under the Dirichlet boundary condition with zero stream function at all the coasts and the open boundaries.

(3) $$\Delta \psi_{i}=\lambda_{i}^{\psi }\psi_{i}\;\mathrm{in}\;\Omega \;\psi_{i}=0\;\mathrm{on}\;\partial \Omega ,$$

where Ω denotes the model domain and ∂Ω denotes the boundaries. The term $\phi_{i}$ is numerically determined by solving the two-order partial differential equation under the Neumann boundary condition with non-normal currents at all of the coasts and the open boundaries.

(4) $$\Delta \phi_{i}=\lambda_{i}^{\phi }\phi_{i}\;\mathrm{in}\;\Omega \;n\cdot \nabla \phi_{i}=0\;\mathrm{on}\;\partial \Omega ,$$

where ***n*** denotes the unit vector perpendicular to the boundaries. The term $\phi_{i}^{b}$ is determined by solving the Poisson equation with a constant divergence over the model domain under the boundary conditions, namely non-normal currents at the coasts and distribution of the normalized current perpendicular to the open boundaries.

(5) $$\Delta \phi^{b}=\int_{\partial \Omega }g_{\phi }(s)ds\;\mathrm{in}\;\Omega \;n\cdot \nabla \phi^{b}=g_{\phi }(s)\;\mathrm{on}\;\partial \Omega ,$$

where any function $g_{\phi }(s)$ can be written as a linear combination of the basis functions $g_{i}(s)$ with amplitudes of 1.

(6) $$\left\{g_{i}(s)\right\}=\left\{1,\;sin(\frac{\pi }{l}s),\;sin(\frac{2\pi }{l}s),\ldots ,sin(\frac{i\pi }{l}s),\ldots \right\},$$

where *s* denotes the coordinate along the open boundary, *l* denotes the length of the open boundary, and *i* denotes the mode number (i=1,2,…). Note that all of the unknown coefficients on the right-hand side of Equation (2) are determined while using the inverse process provided by integral Equation (1).

**Figure 2:**
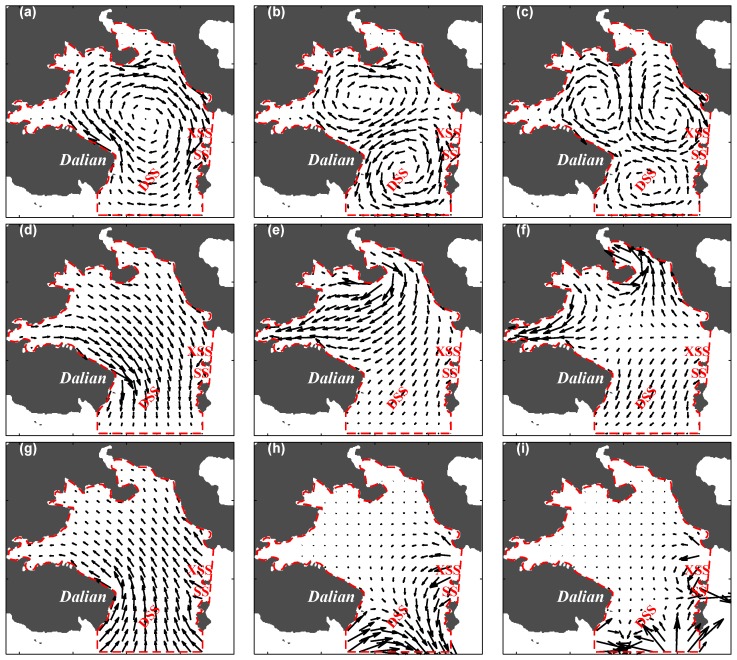
Distributions of the first three modes in the model domain: The first row (**a**–**c**) indicates the Dirichlet modes $\psi_{1}$, $\psi_{2}$, and $\psi_{3}$, the second row (**d**–**f**) indicates the Neumann modes $\phi_{1}$, $\phi_{2}$, and $\phi_{3}$, and the third row (**g**–**i**) indicates the open-boundary modes $\phi_{1}^{b}$, $\phi_{2}^{b}$, and $\phi_{3}^{b}$.

Figure 2 shows the first three modes of the Dirichlet, Neumann, and open-boundary with the vector plots of each current. The first Dirichlet mode produced a clockwise circulation over the bay. The circulation was split into two for the second mode and three for the third mode. The first Neumann mode produced a southward current induced by convergence into the DSS. A converging location was added in the narrow sea near the western coast for the second mode, and was further added in the narrow sea near the northern coast for the third mode. The first open-boundary mode showed convergence toward the northern coasts over the bay and the currents induced by the second and third open-boundary modes were confined around the open boundaries at DSS, SS, and XSS, and minimal effects of the second and third modes occurred at the inner bay.

Substituting Equation (2) into Equation (1), we obtain

(7) $$\Delta \tau =-\frac{2\alpha_{i}^{\phi }}{C_{0}^{2}}\sum_{i=1}^{17}\left[\phi_{i}\left(r_{2}\right)-\phi_{i}\left(r_{1}\right)\right]-\frac{2\alpha_{i}^{\psi }}{C_{0}^{2}}\sum_{i=1}^{17}\left[\int_{0}^{L}k\times \nabla \psi_{i}d\xi \right]-\frac{2\alpha_{i}^{b}}{C_{0}^{2}}\sum_{i=1}^{17}\left[\int_{0}^{L}\phi_{i}^{b}d\xi \right],$$

where $r_{1}$ and $r_{2}$ are the positions of acoustic stations T1 and T2, respectively. Equation (7) is formulated for one transmission path and then extended for the 51 transmission paths. The 17 modes were taken into account for each mode as the best fitting value with the observed RAC data, as presented in the next paragraph. The resulting number of unknown expansion coefficients $\alpha_{i}^{\phi }$, $\alpha_{i}^{\psi }$, and $\alpha_{i}^{b}$ (i=1,2,…,17) became 51. The expansion number of 17 is used for all three modes, because this selection is reasonable in consideration of the horizontal scales to be resolved by each mode. Equation (7) was solved using the tapered least squares method accompanied by the L-curve method [18,19]. The total computational time for the half-day data set is 4 min. for the CFM, and 1 min. for the NCM on a mobile workstation with 2.6 GHz CPU. The time exhausted for the inversion is almost same as that for NCM because one-time calculation of normal modes exhausted 3 min.

**Figure 3:**
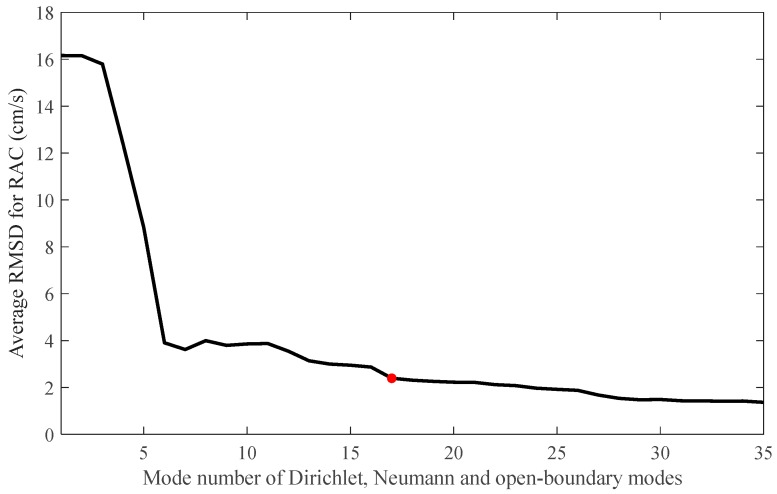
Average root-mean-square-difference (RMSD) of the observed and inverted range-averaged currents (RACs) plotted with respect to the mode number. The red dot indicates the position where the optimal mode number of 17 was determined.

A similar inverse calculation was performed, changing the mode number for each mode. The RMSDs between the observed and inverted range-averaged currents (RACs) were calculated for all of the 51 transmission paths, and the average values are plotted with respect to the mode number in Figure 3. The average RMSD rapidly decreased with the mode number and then reached a small value of 2.4 cm s^−1^ at a mode number of 17. Thus, the inverse calculation was performed in this study while using a mode number of 17.

**Figure 4:**
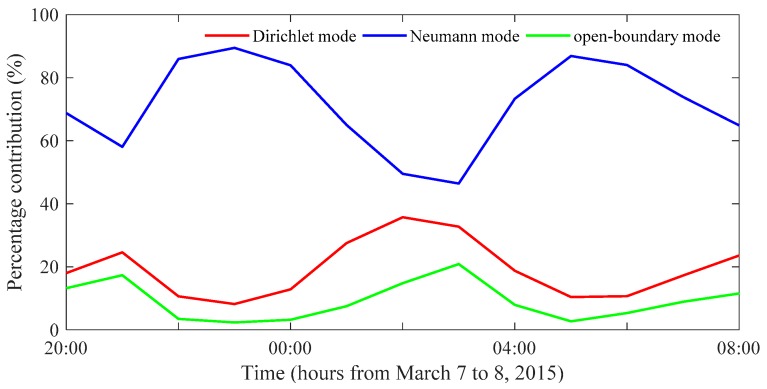
Time plots of the percentage contribution from the Dirichlet mode (red line), the Neumann mode (blue line), and the open-boundary mode (green line) to the total kinetic energy.

The kinetic energy per unit area was calculated from the inverted currents at all of the grid points inside the model domain and then summed over the entire model domain to estimate the total kinetic energy for each mode. Figure 4 plots the percentage contribution from each mode to the total kinetic energy with respect to time. The mean percentage contributions were approximately 70%, 20%, and 10% for the Neumann, Dirichlet, and open-boundary modes, respectively, which indicated that the Neumann mode had the largest contribution. The temporal variation of the Neumann modes was out-of-phase with those of the Dirichlet and open-boundary modes. The predominance of the Neumann mode is caused by that the major model domain, except for regions near the coast that are dominated by tidal currents in an inviscid fluid. Additionally, the viscous and inviscid components corresponding to the Dirichlet and Neumann modes, respectively, are traded off over the whole model domain.

## 3. Inverted Currents

**Figure 5:**
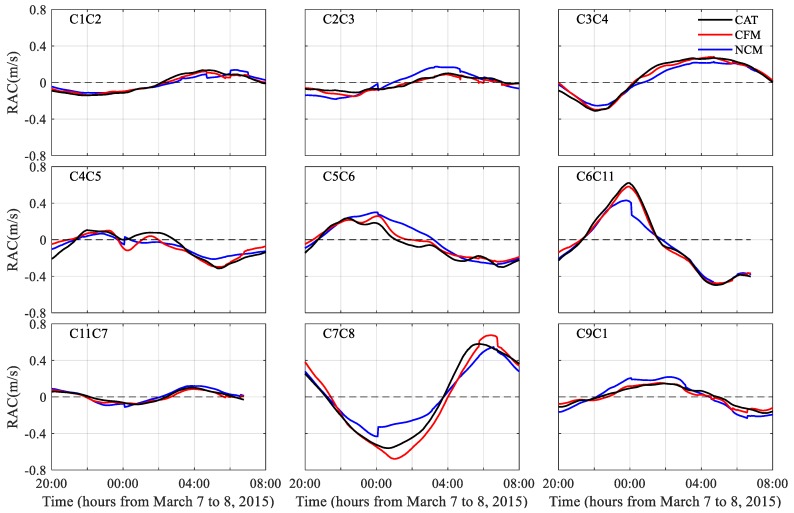
Comparison of RACs that were obtained by coastal acoustic tomography (CAT) observation (black line), coast-fitting method (CFM) (red line), and no coast method (NCM) (blue line) for the nine transmission paths at the periphery of the tomography domain.

RACs calculated while using the CFM and NCM were compared with the observed RACs that were obtained along the nine peripheral transmission paths (Figure 5). For all of the transmission paths, except for C1C2 and C11C7 with good agreement between the two types of data, the inverted RACs were significantly closer to the observed RACs for the CFM than for the NCM. Table 1 presents a comparison of the RMSDs between the observed and inverted RACs. The mean RMSDs for all the nine peripheral RACs were 3.9 cm s^−1^ and 7.2 cm s^−1^ for the CFM and the NCM, respectively. The mean RMSDs for all the RACs decreased to 2.2 cm s^−1^ and 3.7 cm s^−1^ for the CFM and NCM, respectively. The peripheral-line data mainly cause the improvement of the data quality by the CFM.

**Figure 6:**
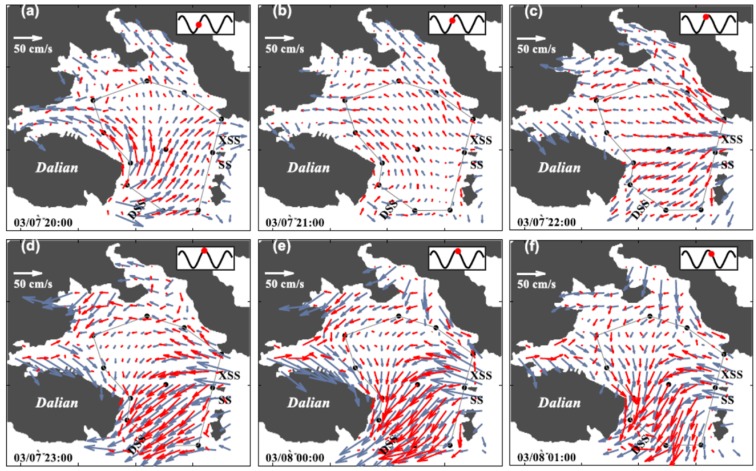

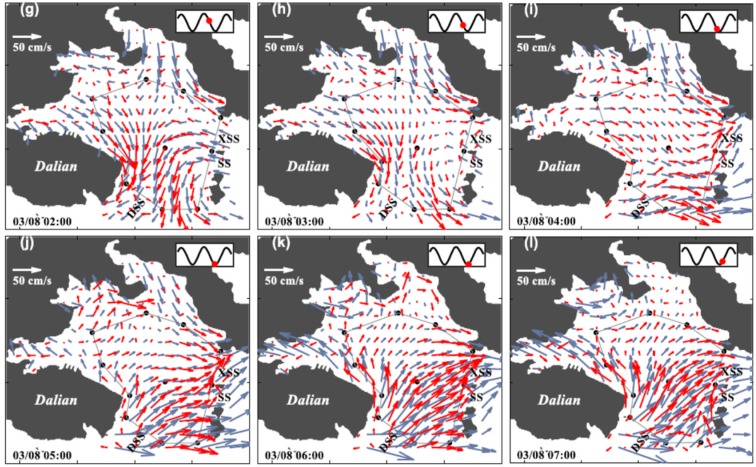
Hourly mapping (**a**–**l**) of the hourly-mean horizontal current fields calculated using the CFM (red arrows) and NCM (gray arrows). The observation time and the corresponding sea level height (red dot) are indicated in the lower-left and upper-right corners, respectively.

**Figure 7:**
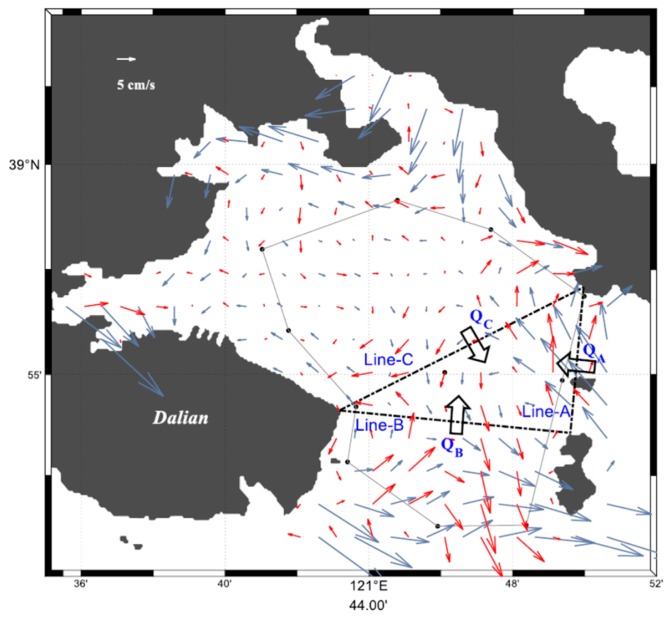
Distribution of the 12-h mean residual current. The red and gray arrows indicate results obtained from the CFM and NCM, respectively.

A comparison of the distribution of the hourly mean inverted currents obtained while using the CFM and the NCM during half day from 20:00 on 7 March to 08:00 on 8 March 2015 is shown in Figure 6 with the vector plots. The inflow/outflow for the inner bay occurred at flood/ebb tides with a range of 0.2–0.3 m s^−1^ (Figure 6a,b,g,h). The strong currents with a range of 0.4–0.5 m s^−1^ passed through the bay mouth southwestward at high water (Figure 6d,e) and northeastward at low water (Figure 6j,k), which produces slight inflow and outflow at the inner bay. For the CFM results in particular, significant along-coast currents occurred in the near-coast region north of transmission path C1C2, reaching 0.2 m s^−1^ westward/eastward at high/low water. The unnatural NCM-derived currents crossing the northern coast of Dalian City diminished in the CFM-derived currents (Figure 6c–g). The residual current that was calculated by taking the average of the 12-h data from 20:00 on 7 March to 08:00 on 8 March 2015 is shown in Figure 7 with the vector plot. The CFM-derived residual currents reached 0.1 m s^−1^ at the bay mouth and 0.05 m s^−1^ in the near-coast region of the northern part of the bay. The NCM-derived residual current was overestimated, particularly in the near-cost regions outside the tomography domain. Slight residual currents occurred in the central region of the inner bay for both the CFM and NCM results.

## 4. Error Estimate

**Figure 8:**
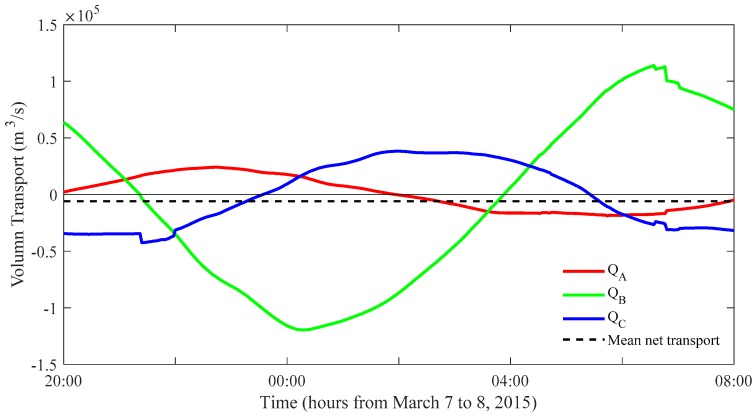
Volume transport variations for *Q_A_* (red line), *Q_B_* (green line), and *Q_C_* (blue line) during half day from 20:00 on 7 March to 08:00 on 8 March 2015. The dashed line indicates the 12-h average, ΔQ.

The error velocity for the semidiurnal tidal currents was estimated from the RMSDs between the observed and inverted RACs while using the hourly mean data. As presented in Table 1, the RMSDs averaged over all of the transmission paths were 2.2 cm s^−1^ and 3.7 cm s^−1^ for the CFM and the NCM, respectively. The volume transports, *Q_A_*, *Q_B_*, and *Q_C_* across the triangular domain surrounded by Line-A, Line-B, and Line-C at the bay mouth were calculated to evaluate the net volume transport, ΔQ crossing the triangular domain while using the following formula (see Figure 7):

(8) $$\Delta Q=Q_{A}+Q_{B}+Q_{C},$$

where positive transport is inflow into the triangular domain. The temporal variations of the hourly mean, *Q_A_*, *Q_B_*, and *Q_C_* are shown in Figure 8, together with the 12-h average of ΔQ. *Q_A_* and *Q_B_* showed an out-of-phase relation with significant difference in the amplitude. *Q_C_* showed that the inflow into the inner bay mainly occurred during flood tide and outflow from the inner bay occurred mainly during ebb tide. The 12-h average, ΔQ of 0.06 × 10^5^ m^3^ s^−1^ and the vertical section area of 6.8 × 10^5^ m^2^ along Line-C resulted in a vertical section average current of 0.9 cm s^−1^, which corresponded to the error velocity for the residual current.

## 5. Conclusions

In this study, tomographic inversion (the CFM) that was based on the function expansion using three types of coast-fitting normal modes (the Dirichlet, Neumann, and open-boundary modes) was used to reconstruct the data set that was obtained in the 2015 Dalian Bay experiment with 51 transmission paths. The optimal mode number of 17 was determined at a position on the mode number-RMSD diagram, where the RMSD between the observed and inverted RACs for the nine peripheral transmission paths was as small as 2.4 cm s^−1^. The inverted current fields that were obtained by the CFM were in good agreement with the results that were obtained while using the conventional NCM at the central part of the tomography domain. The CFM-derived currents significantly improved around the peripheral transmission paths for the NCM with a constraint that only one transmission path is available around the peripheral path. This constraint for the NCM was reduced in the CFM, owing to the dynamic interpolation by the normal modes. Furthermore, the dynamic interpolation provides reasonable current fields in the near-coast regions outside the tomography domain. The error velocities were 2.2 cm s^−1^ for the semidiurnal tidal current and 0.9 cm s^−1^ for the residual currents, which are significantly smaller than the amplitude of each current.

It is concluded that the CFM is suited to tomographic inversion for semi-enclosed bays.

## Figures and Tables

**Table 1:** Comparison of RMSDs for the RACs between the CAT-observed and CFM-derived currents, and between the CAT-observed and NCM-derived currents.

RMSD (cm/s)	CFM	NCM
C1C2	1.9	3.7
C2C3	2.1	6.8
C3C4	2.9	6.0
C4C5	6.9	6.0
C5C6	4.8	10.8
C6C11	3.6	10.3
C11C7	1.5	2.7
C7C8	9.2	13.2
C9C1	2.3	5.6
Peripheral mean	3.9	7.2
All data mean	2.2	3.7
